# Mobility census for monitoring rapid urban development

**DOI:** 10.1098/rsif.2023.0495

**Published:** 2024-05-08

**Authors:** Gezhi Xiu, Jianying Wang, Thilo Gross, Mei-Po Kwan, Xia Peng, Yu Liu

**Affiliations:** ^1^ Institute of Remote Sensing and GIS, Peking University, Beijing, People’s Republic of China; ^2^ Centre for Complexity Science and Department of Mathematics, Imperial College London, London, UK; ^3^ Institute of Space and Earth Information Science, The Chinese University of Hong Kong (CUHK), Hong Kong, People’s Republic of China; ^4^ Helmholtz Institute for Functional Marine Biodiversity (HIFMB), Oldenburg, Germany; ^5^ University of Oldenburg, Institute of Chemistry and Biology of the Marine Environment (ICBM), Oldenburg, Germany; ^6^ Alfred-Wegener Institute, Helmholtz Center for Marine and Polar Research, Bremerhaven, Germany; ^7^ Tourism College, Beijing Union University, Beijing, People’s Republic of China

**Keywords:** human mobility, manifold learning, cities

## Abstract

Monitoring urban structure and development requires high-quality data at high spatio-temporal resolution. While traditional censuses have provided foundational insights into demographic and socio-economic aspects of urban life, their pace may not always align with the pace of urban development. To complement these traditional methods, we explore the potential of analysing alternative big-data sources, such as human mobility data. However, these often noisy and unstructured big data pose new challenges. Here, we propose a method to extract meaningful explanatory variables and classifications from such data. Using movement data from Beijing, which are produced as a by-product of mobile communication, we show that meaningful features can be extracted, revealing, for example, the emergence and absorption of subcentres. This method allows the analysis of urban dynamics at a high-spatial resolution (here 500 m) and near real-time frequency, and high computational efficiency, which is especially suitable for tracing event-driven mobility changes and their impact on urban structures.

## 1. Introduction

Understanding the dynamics of cities is a central goal of urban studies. A variety of data-driven models have offered insights into the evolution of urban structures, focusing on diverse socio-economic observables including income inequality [1,2], ethnic identities [3,4] and environmental impact [5,6]. For example, polycentric transitions are conceptualized as outcomes of competition between areas, defined by their economic allure and traffic congestion [7]. Similarly, urban scaling laws have been delineated through a balance between socio-economic outputs and infrastructural costs [8].

Traditionally, the development of cities has been studied using a variety of methods and data sources, including census datasets [9–11]. For instance, decadal censuses, such as the UK census, provide comprehensive information on an array of social variables such as education outcomes, employment status and housing conditions, gathered from population surveys and aggregated spatially. Complementary to these are non-census datasets, including the American Communities Survey [12] and the UK’s Indicators of Multiple Deprivations [13]. Despite offering high-quality data from exhaustive surveys, the significant cost and time involved mean that census and similar datasets are released at long-time intervals, thus offering only periodic snapshots of urban evolution. Additionally, relying on predetermined question catalogues makes these types of data less effective in identifying unanticipated developments.

To uncover emergent developments, analysis of real time and alternative data sources is desirable. For instance, Germany has utilized open-source mobility data to analyse social structures and contact patterns during the COVID-19 pandemic [14]. The introduction of high-frequency mobility data has enabled rapid analysis using unstructured and noisy, yet rich and comparatively unbiased, datasets, revealing the critical and diverse urban structures on much shorter timescales, e.g. the spatial and temporal decomposition of visitation [15], the impact of cultural ties on human mobility [16], and the nexus between contact patterns and epidemic propagation [17,18].

Mobility datasets are an incidental by-product of our modern interconnected society. For example in mobile communications mobility traces are produced as a by-product of the normal operations of network providers. Because the movement of individuals often occurs as a result of social needs, mobility data contain a wealth of information on social geography. However, as these data are not produced for this purpose, it only implicitly contains the social information. Careful data analysis is therefore required to extract salient social variables from mobility traces.

In the analysis of tabular census-like datasets, recent progress has been made using diffusion maps [19,20], a manifold learning technique that reduces the dimensionality of structured datasets in biological and social studies [21–24]. Diffusion maps provide a nonlinear, deterministic and hypothesis-free approach that pinpoints explanatory parameters in large high-dimensional datasets. For example, diffusion maps were recently used to extract explanatory variables from census data of specific cities and countries [24,25], spotlighting higher education and deprivation hubs as key factors shaping their urban environment. The idea behind manifold-learning methods such as the diffusion map is that current datasets record much more information than is necessary to encode the salient information. The diffusion map can therefore reduce the number of variables by identifying the main variables that are needed to span the variation of data in the dataset.

Here, we propose the mobility census (MC), a computational framework for high-frequency analysis of urban structure. We start with a dataset of mobility traces that we segment into a 500 m spatial grid. For each grid cell, we then compute a set of 1665 different *mobility variables* from the available traces. We work on the assumption that if a sufficiently large catalogue of such variables is computed then the desired social information will become encoded in the resulting data table. We then use diffusion mapping (DM) to reduce the dimensionality again and extract a set of aggregated variables that account for the majority of the variance between cells and thus make the social information accessible in distinct variables.

Using multi-year high-frequency mobility data from Beijing as an example, we discover the polycentric isolation patterns and separate local and global mobility features by analysing indicators. Using additional data, we can interpret the eigenfeatures (EFs) found by the diffusion map, and identify economic prosperity, location and local irreplaceability as the most important mobility variables. Furthermore, we trace Beijing’s accelerated evolution, including the evolution of subcentres from the functional supplements of the main city to independent entities. In some instances, this transformation can be attributed to substantial events like new airport construction, while in others, it is the cumulative effect of numerous smaller-scale changes. Thus, this study captures the dynamics of modern urban environments, paving the way for more nuanced understandings of city structures.

## 2. A census for human mobility

The MC method is a productional generalization of manifold learning by setting up a protocol first to aggregate the individual trajectories through each small area into a table of ‘mobility variables’, then to apply DM analysis to map the urban structures through DM EFs. The method is based on simple intuitions: a limited number of functional place categories influence human movements. Hence, these categories should become encoded in movement traces, and thus can be extracted by suitable analysis.

To show the application of the MC method, we use a dataset containing movements of all China Unicom subscribers in Beijing from 1–31 August 2018, and 1–31 May 2021, amounting to *ca* 11.57 × 10^6^ users and 1.8 × 10^9^ trips, where a trip is an individual’s single visitation from an origin to a destination. China Unicom is one of the three major ICT providers in China and Beijing, whose trip data have provided insights into many socio-economic aspects such as tourism and local imbalanced developments [26–29]. We note that already one month of data is sufficient to reveal key elements of the evolving urban structure (see below). Moreover, we verified that the coverage rate of the China Unicom does not have a significant spatial bias in terms of districts (see electronic supplementary material, figure S1), and thus should provide a reasonably unbiased view of the spatial structure of the city.

**Figure 1.**
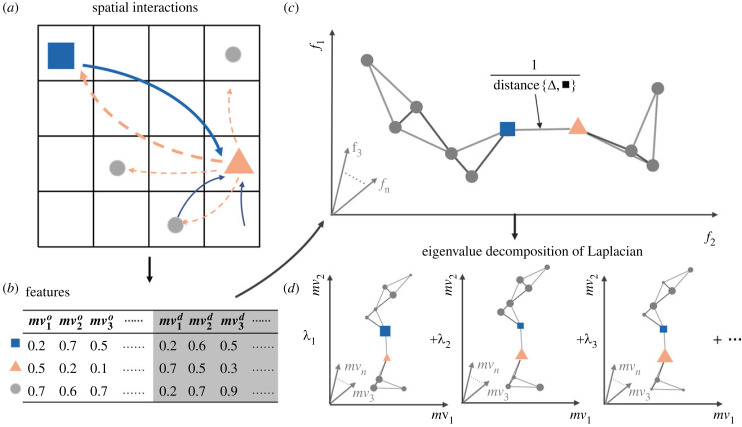
Sketch of the mobility census framework. The blue squares, grey circles and orange triangles label the cells, while the arrows represent human mobility flows. The widths of these arrows denote the frequency of visits. (*a*) Flow data are aggregated in a high-resolution grid with temporal and spatial granularity of 1 h and 500 m, respectively. (*b*) A large set (here 1665) of statistical properties are calculated for each grid cell for each hour of the day, resulting in a high-dimensional data table. This table is then aggregated to provide a monthly summary of mobility patterns. (*c*) To reduce the dimensionality the diffusion map is used, which is the first step constructing a network in which each spatial cell is connected to the *k* most similar cells, and links are weighted by the respective distance. (*d*) Finally, eigenvectors of a Laplacian matrix describing the data are computed. These eigenvectors assign new variables to the cells, providing a meaningful low-dimensional dataset parameterization. Subsequently, common analysis tools can be applied to this representation of the city.

We partition the area of Beijing by a grid of 500 × 500 m cells (number of cells *n* = 22 704). For each cell, movements originating or concluding within it are identified, resulting in variable-length lists of timestamped movements (figure 1*a*). These movements are then represented in the form of an origin–destination matrix for each respective hour, organized by both origin and destination cells. Acknowledging the potential influence of the modifiable areal unit problem (MAUP) on our results, we performed a sensitivity analysis by re-partitioning the area into a 1 × 1 km grid, and compared the results derived from the 500 m and 1 km grids. This sensitivity analysis showed that our primary observations were consistent across different grid sizes. However, the impact of more localized, detailed activities and the significance of specific, less obvious patterns (e.g. neighbourhood-wise home-work segregation that is distinct within a 1 km scale) varied with the change in grid size. The larger grid analysis mostly confirmed our initial findings at 500 × 500 m cells, particularly for broad spatial patterns like commuting and night-time activities. However, it also highlighted finer distinctions in small-scale patterns such as the delineation of residential and work areas. Despite these differences, our main findings based on the 500 m grid remained robust, illustrating the general properties of human mobility and nuanced patterns at a community scale (500 m).

To reduce the complexity of the dataset, we aim to identify the essential *features* and their combinations that shape human movements. We collect characteristic statistical attributes indicative of an area’s movement, hereinafter referred to as *mobility variables* (figure 1*b*). These mobility variables cover a full range of topics from existing literature that associates the movement properties with urban developments, e.g. the number of trips originating from the area, the total distance of all trips, or the average speed of movement within the area on weekdays. Each of the mobility variables quantifies the collective traits of the trips that start or terminate in the respective cell (precise definitions of the mobility variables in electronic supplementary material, table S1). Furthermore, we incorporate certain geostatistical operators (e.g. the H-index and Gini coefficient, see electronic supplementary material) to the basic statistics to cope with human mobility partially driven by the places’ comprehensive functions [30]. The additional operators help to reveal the nonlinear responses of location attractiveness to human movements. In this manner, we generate a census-like feature table, offering a fixed dimensionality of 1665 mobility variables for each cell. This breadth of mobility variables prevents over-reliance on a limited set of variables, which is particularly important in complex urban settings.

Constructing the feature table (figure 1*b*) brings structure and a first reduction in data complexity, but the feature table is still a high-dimensional dataset that suffers from the curse of dimensionality [31]. To recover the most dominant factors determining the attractiveness of locations, we then explore this table using diffusion map analysis (figure 1*c*,*d*) that was previously applied to census data [24]. The basic idea of the diffusion map [19] is that salient features of the data can be discovered by analysing the topological structure of the dataset. A central insight underlying the diffusion map is that comparisons between very dissimilar objects are highly unreliable and introduce noise that can quickly swamp the salient information. It is therefore essential to remove such low-confidence comparisons of cells from the analysis. The analysis starts by finding the most similar pairs of cells. Following [22], we compute the similarities between cells as a Spearman rank correlation [32] between the cell’s feature list (see electronic supplementary material). Utilizing a proven approach [33,34], we limit the comparisons used in the subsequent steps to the 10 most similar cells of each cell.

The remaining comparisons of mobility features between cells now form a complex network (figure 1*c*) that can be mathematically described by a row-normalized Laplacian matrix [24]. The dimension of the eigenvectors of this matrix equals the number of cells (figure 1*d*). Hence, each eigenvector of the Laplacian assigns a value to each of the cells. We can thus interpret the entries of each of the eigenvectors as a new feature for the cells. The features identified in this way are in many ways similar to principal components [35], but provide a more robust, nonlinear parameterization of complex high-dimensional data.

**Figure 2.**
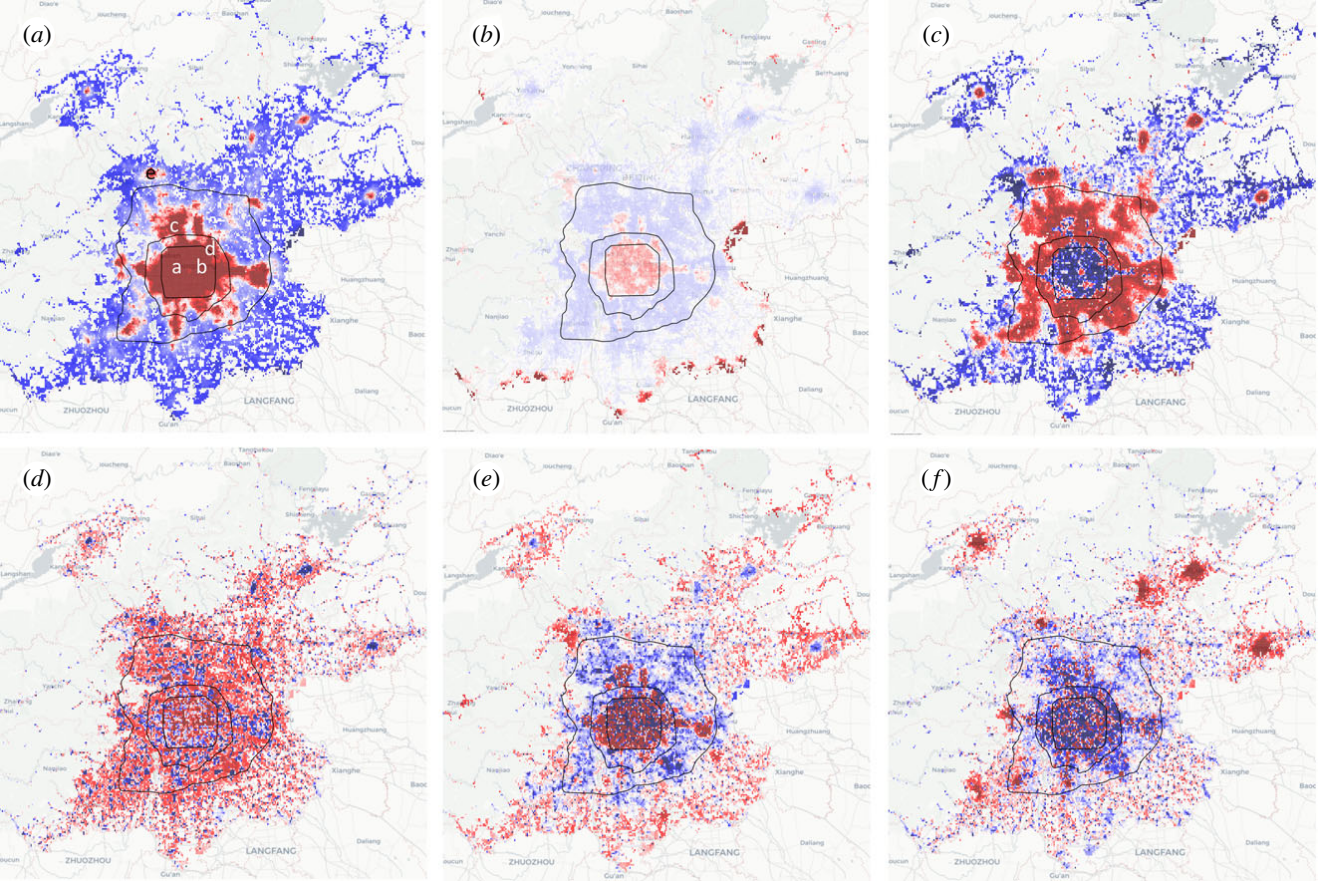
Explanatory variables (EF) identified by the mobility census are depicted in (*a*–*f*), presented in descending order of importance. Values are represented by a gradient where shades of red correspond to more positive entries and shades of blue denote more negative entries. We interpret the EFs operationally as indicators of centrality (*a*), entry points to the city (*b*), local heterogeneity (*c*), livability (*d*) workplaceness (*e*) and attractiveness for long-distance journeys (*f*). Labelled places in (*a*) are Financial Street (a), China World Trade Centre (b), Xierqi–Huilongguan subdistrict (c), Wangjing subdistrict (d) and Changping town (e). These demonstrate that DM can identify informative features in the data.

In the following, we refer to the new features identified from the DM as EFs. Each EF corresponds to an eigenvalue that scales inversely with the variation captured by the respective feature. Hence the eigenvalues are indicative of the importance of the respective features, such that the most important EF is the one with the lowest non-zero eigenvalue. We note that the DF analysis identifies important statistical patterns but does not provide an interpretation of these patterns. Instead, we use two approaches to help us formulate hypothesis regarding these patterns: first we can visualize important EFs on a map by colour-coding grid cells according to the value of the respective EF (figure 2). Second, we correlate the EFs with the original mobility variables to identify the mobility variables to which a particular EF is linked.

## 3. Dominant patterns

We start our analysis by plotting the most important EFs of Beijing that were derived from the mobility data of the year 2021, (figure 2) colour-coded by the EF entries, and calculate the correlation between the EFs and the original mobility variables (most correlated ones in table 1). We claim that most interpretations of the EFs are consistent between the MC of the year 2018 and 2021 with few exceptions, primarily due to pronounced event-driven changes in visitations (discussed further in §4). This provides some indication of the robustness of the MC results.

**Table 1.** Correlations between mobility variables and diffusion map eigenvectors. Gini(·) represents the Gini coefficient applied to a variable within its 2 km neighbourhood. LR(·) denotes the ratio of a cell's variable value to the mean value of that variable across the cell’s 2 km neighbourhood. K is the kurtosis of the distribution. The notation (w, p1) is the first percentile of a characteristic of a cell as destination, while ·(h, p2) is the second percentile of a cell’s characteristics as origin. (*t*) Specifies the mobility metric at *t* hours since midnight.

rank	*f*_1_	corr	*f*_2_	corr	*f*_3_	corr
1	H-index (commute)	0.8843	Gini (flow ratio (16))	0.7238	Gini (H-index)	0.5636
2	out-flow (15)	0.8621	Gini (flow ratio (12))	0.6957	Gini (ROG(w, p4))	0.5330
3	out-flow (12)	0.8613	Gini (flow ratio (17))	0.6810	Gini (ROG(w, p3))	0.5300
4	out-flow (16)	0.8610	Gini (stay duration (h, p8))	0.6763	Gini (ROG(w, p5))	0.5245
5	out-flow (total)	0.8605	Gini (flow ratio (13))	0.6691	Gini (travel Dis(w, p6))	0.5132
rank	*f*_4_	corr	*f*_5_	corr	*f*_6_	corr
1	LR (in-flow (10))	0.8419	net commute flow	0.4989	average travelling time (w, p4)	0.2892
2	LR (r-population)	0.8265	in-flow (8)	0.4943	average travelling time (w, p5)	0.2881
3	LR (in-flow (2))	0.8190	in-flow (9)	0.4873	average travelling time (w, p3)	0.2839
4	LR (in-flow (21))	0.8177	entropy of work	0.4588	average travelling time (w, p6)	0.2797
5	LR (in-flow (20))	0.8175	LR (in-flow(8))	0.4451	average travelling time (w, p2)	0.2726

For the first EF, *f*_1_, we find the highest values in the centre, which coincide with central business areas such as Financial Street (a), China World Trade Centre Towers (b) and Sanlitun. Pronounced local maxima also occur at emerging hubs of economic activity such as Xierqi-Huilongguan figure 2*a*(c) and Wangjing, figure 2*a*(d), well known as the headquarters of most high-paying, high-tech companies, which act as local hubs of development. We thus conclude that *f*_1_ detects a high density of workplaces in the urban centre and subcentres.

To further explore *f*_1_, we find the most strongly correlated mobility variables. The strongest correlations with indicators of a high volume of flow toward areas ranked highly by *f*_1_ (0.86, *p* < 0.001). This is consistent with our interpretation as the 1% of cells that score highest in this indicator contains 6% of the residential population but 13% of the workplaces. Also highly correlated is an indicator of flow diversity (0.57, *p* < 0.001), which indicates that the areas highlighted by *f*_1_ receive flow from a diverse range of origins.

The second EF, *f*_2_, is the most strongly localized of the first six EFs, which can be mathematically verified by computing the inverse participation ratio (see electronic supplementary material). It has pronounced maxima at several locations in the south where major highways, such as the G103, G106, G230 and G102, enter the city. Moreover, we find a maximum in the centre of Beijing at Sanlitun, an area well known for its embassies and nightclubs. What unites these locations is that they receive significant long-distance travel during night-time hours, which is due to late-night partygoers (Sanlitun), or trucks, which are not allowed to travel in the daytime under Chinese regulations (motorway entry points). The long-distance, night-time visits create a distinct traffic pattern that the DM picks up. The most correlated variable with *f*_2_ is an indicator of the diversity of in- and out-flow (0.72, *p* < 0.001) and trip duration (0.68, *p* < 0.001), which is consistent with this interpretation.

In 2018, *f*_2_ also highlighted some areas in the northern subcentres (see electronic supplementary material), but the respective maxima are no longer visible on the map for 2021. It can be interpreted as a sign that the subcentres have lost attractiveness as long-distance destinations in this period. Indeed, the house–job ratio and residential population in these subcentres increased significantly [36] and hence likely receive less long-range commuter traffic.

EF *f*_3_ has a pronounced concentric structure, with strong positive values found both in the city centre and outlying villages, whereas the outer areas of the city are assigned negative values. In diffusion maps, such high–low–high patterns can appear as harmonic modes of other prominent features. One must therefore be particularly careful to avoid over-interpreting them. However, in a real date, even harmonic modes often convey useful information.

Considering the metrics that correlate with *f*_3_ highlight an indicative measure, which we refer to as the ‘diversity of centrality values,’ mathematically represented by the Gini coefficient, calculated on the h-index, where the h-index is defined as the count of destinations in a target cell’s neighbourhood that each have a flow volume exceeding *h*. This indicator underscores the variation in the importance, or centrality, of the neighbouring destinations around a particular location. Thus places receiving high values in this EF are those surrounded by locations that differ in importance. Such differences are very pronounced in the city centre, whereas the outer areas supporting the centre are much more uniform. In the outermost belt, strong differences return, likely due to the spatial self-organization of outlying villages [37]. Hence, the boundary line where *f*_3_ crosses from the negative back into the positive can be regarded as the true boundary of the city.

**Figure 3.**
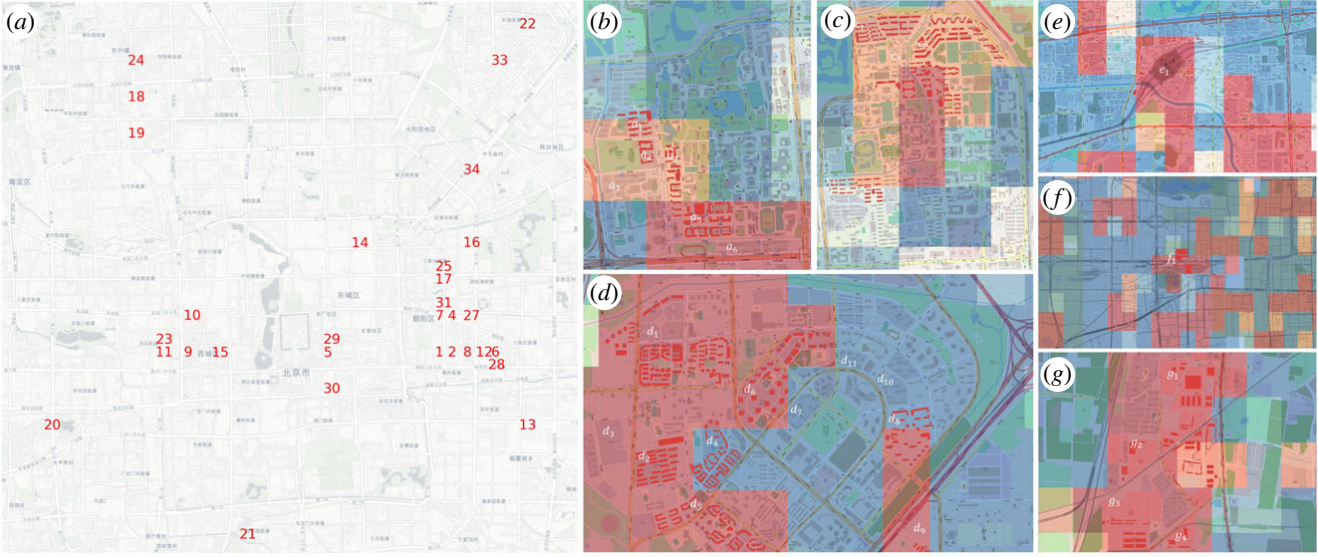
Small-scale patterns of the EFs determined by the spatial transitions of extreme values of EF. (*a*) Blue cells correspond to the largest 100 entries of the differences *f*_3_ − *f*_1_, in the central part of Beijing (Fifth Ring Road). Red numbers are top-searched shopping malls retrieved from Google Map API. (*b–d*) Peking University, Tsinghua University and Wangjing. Cells are coloured by the entries of *f*_5_ as in figure 2*e*, thus red for positive entries and blue for negative entries. Low transparent red and blue highlight the uses of buildings, such as dormitory/residential (red), and teaching/office buildings (blue). (*e–g*) South and West Railway Station, and Xinfadi wholesale food market, coloured by the entries of *f*_6_ from the most negative (blue) to the most positive (red). High values of *f*_6_ correspond to areas that are visited by visitors from distant origins. Specific labels of locations are listed in electronic supplementary material, table S3. These results illustrate that the mobility census reveals some insights down to the 500 m scale.

Computing the difference between the *f*_3_ (indicating variance of centrality) and *f*_1_ (indicating centrality) highlights local places of interest. To confirm this, we compared the highest values of *f*_3_ − *f*_1_ in the centre to the most searched shopping malls which reveals very good agreement (figure 3*a*).

The next three EFs have a pronounced structure on the 500 m scale. EF *f*_4_ correlates very well with sinks and sources of short-range commuter traffic, with positive (negative) values marking the sources (sinks) of flows (figure 2*d*). The EF also correlates strongly with mobility variables that measure the relative volume during hours that correspond to typical closing times of businesses, corroborating this interpretation (e.g. with correlation coefficient 0.82, *p* < 0.01 with in-flow at 20.00). We see that emerging software industry centres at Xierqi, Wangjing and Yizhuang all receive strongly positive values. EF *f*_5_ is similar but correlates with morning opening hours rather than evening closing times (e.g. with correlation coefficient 0.49, *p* < 0.01 with in-flow at 20.00). These observations suggest *f*_4_ and *f*_5_ being livability and workplaceness indicators, respectively.

To corroborate the interpretation of *f*_5_, we also explored it on a smaller scale by considering the locations of Peking University, Tsinghua University and Wangjing. These locations are identified by the largest average differences of the *f*_5_’s entries with their neighbours. The detailed scale *f*_5_ separates workplaces and residential areas within these areas 3*b*–*d*.

EF *f*_6_ also exhibits a highly detailed pattern with positive and negative values occurring often in close proximity. However, the centre receives mostly positive values, whereas the subcentres have mostly negative entries. This EF correlates strongly with mobility variables indicating long-distance trips. Hence we interpret this EF as an indicator of long-distance attractivity. It is confirmed by considering the entries on the detailed scale where the highest values of this EF are found at railway stations and the largest wholesale food market (figure 3*e*–*g*).

Interestingly, repeating the analysis for 2018 (electronic supplementary material, figure S10) also reveals pronounced positive values in the sub-centres, which have vanished by 2021. It could indicate a change in mobility behaviour induced by the COVID-19 restrictions, which also constrained travel on this scale and/or the increasing residential population mentioned above.

## 4. Subcentre evolution

Above we showed that the DM can extract salient functional variables (the EFs) from the high-dimensional set of mobility variables. It thus provides a reduction of the dimensionality of the data that is also valuable for subsequent analysis. The EFs effectively reduce the dimensionality of our data and highlight key mobility patterns within the city. However, each EF represents a specific aspect of urban mobility, and considering them individually might not provide a complete or coherent picture of the overall structure of the city. Additionally, the sheer number of EFs can make it challenging to identify overarching patterns or to compare different regions of the city.

Here, we further aggregate the data by applying a Gaussian mixture model (GMM) [21], a statistical model, that can be used to break the data into distinct clusters. In the Beijing data, GMM identifies six clusters (see electronic supplementary material) representing six types of areas distinct by similar mobility properties.

**Figure 4.**
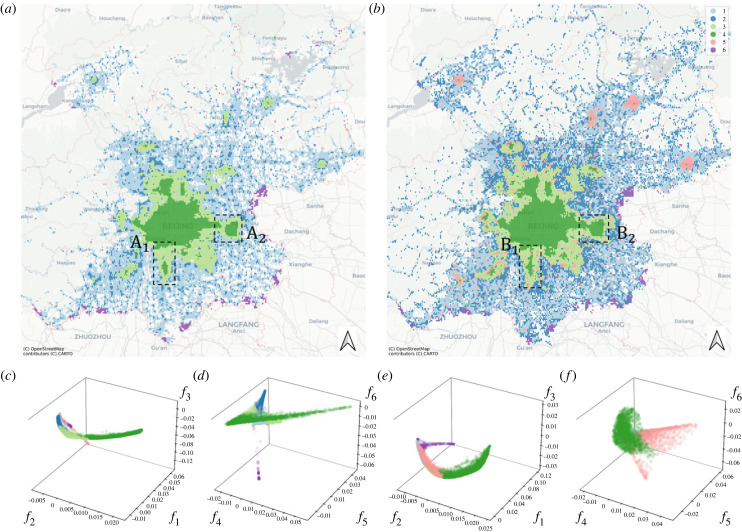
Classification of areas and urban development. Clustering results are displayed both on maps (*a*,*b*) and in a data space spanned by three of the EFs, EF 1, 2 and 3 (*c* for 2018 and *e* for 2021) and EF 4, 5 and 6 (*d* for 2018 and *f* for 2021). Results are shown for one month in 2018 (*a*,*e*,*f*) and one month in 2021 (*b*,*c*,*d*). Labels indicate Daxing (1) and Tongzhou (2) on the maps. The results show that nicely coherent clusters are obtained (colours 1–6), which identify distinct functional areas of the city. The longitudinal comparison illustrates the emergence of distinct subcentres in the north and the absorption of subcentres at Daxing and Tongzhou.

To gain a visual impression of the quality of the clustering result, we can visualize the clusters in the data space defined by the most important EFs (figure 4*c*–*f*). This visualization shows the partition of the data manifold into coherent sections. Colouring the clusters in geographical space (figure 4*a*,*b*) reveals a clear separation into different areas, which we can operationally identify as rural areas (clusters 1,2, depending on local centrality), urban fringe (3), urban centre (4), subcentres (5) and major gateways to the city (6).

We now use these operational designations in a longitudinal comparison of the situation in 2018 and 2021. While the big picture in both of these years is similar, there are some notable differences. First, we note that the category that we identified as a subcentre is much more prominent in 2021 than it was in 2018. During this period, three areas in northeastern Beijing (Pinggu, Huairou and Miyun) and one area in northwestern Beijing (Yanqing) transitioned from the urban fringe to the subcentre category. We conclude that new work opportunities, a rising residential population, and also possibly COVID-19-related mobility restrictions have caused these four areas to develop into fully fledged subcentres, which was also confirmed by field research from the literature [38,39].

Another area of interest is Daxing in southern Beijing (*A*_1_ in figure 4*a* and *B*_1_ in figure 4*b*). In 2018, this well-developed subcentre is classified as an urban centre while being separated from the main city centre by an area of the urban fringe. By contrast in 2021, an area of 39 grids (approx. 9 km^2^) that covers the central area of Daxing has become connected to the main city centre. A major event in this area that occurred in the intervening period is the opening of Beijing Daxing International Airport. We conclude that the construction and opening of this airport tied Daxing closer to the city centre, which is also evidenced by the construction of major motorways and underground connections in this area. As a result, the subcentre of Daxing was effectively absorbed into the city centre.

We see a similar development also in Tongzhou (43 differently classified cells from *A*_2_ in figure 4*a* to *B*_2_ in figure 4*b*, approx. 10.75 km^2^), which becomes likewise connected to the city centre between 2018 and 2021. In this case, the development was likely triggered by the relocation of the Beijing municipal government to Tongzhou in 2019.

## 5. Conclusion

In this paper, we proposed a new method, the MC, for the analysis of urban structure from big unstructured datasets. The proposed method first generates a large number of different metrics (here 1665 mobility variables, elaborated in electronic supplementary material, section II) for each geographical area, to turn the unstructured dataset into a structured table. We then use the diffusion map to extract a smaller number of salient features. This reduces the dimensionality of the data, and thus avoids the ‘curse of dimensionality’ while enabling subsequent analysis. Beyond the particular application considered here, other unstructured data sources could be analysed using the same approach: breaking the domain of interest into small units, compiling a large table of statistical features for these units, and using DM to extract comprehensive features.

The primary limitation of the MC is its focus on active individuals, neglecting the city’s vulnerable groups. This oversight can lead to a skewed understanding of urban dynamics, as it fails to capture the mobility challenges of less mobile or less connected populations such as the elderly, disabled or economically disadvantaged.

By contrast, the major advantages of the MC are that it can reuse data that is already available, reducing costs and workload. It provides results very fast on a near-real-time basis, requiring few weeks of data and negligible processing time, which opens up the option to keep pace with urban development while it happens. Finally, it avoids reliance on a narrow question catalogue, which enables the discovery of novel features not anticipated by the researcher.

Application of the MC to Beijing showed that the method can identify distinct functional classes of areas. While the diffusion map does not in itself provide an interpretation of these classes, interpretations can be assigned using expert knowledge. We note that such interpretations, including those in this paper, should at first be treated as hypotheses, but can later be corroborated using additional analysis and data.

Here, this analysis identifies major explanatory variables that shape the city (cf. [24]), such as attractivity, workplace/housing density and night-time activity. Revealing these features provides insights into the functional organization of cities and their temporal evolution. Notably, the method provides this information with high spatial (here, 500 m) and temporal (hourly basis, aggregated to one-month collection of mobility variables) resolution.

The dimensionality reduction provided by the diffusion map also enables subsequent steps, such as the clustering analysis presented here. We showed that this analysis provides a useful tool to categorize areas within cities and identify boundaries. Moreover, it provides a high-resolution view of important geographical processes, such as the emergence of fully fledged subcentres and the absorption of subcentres into the city centre.

The mobility data used in this study are presently produced on a massive scale as a by-product of mobile communication. The MC method can be applied to aggregated data products of such mobility data, thus avoiding data protection concerns. Moreover, it provides a numerically efficient, deterministic and hypothesis-free approach to the analysis. We envision that in the future, the application of this method may provide a high-resolution and near-real-time view of the evolution of our ever-growing and ever-accelerating urban environments.

## Data Availability

The code to derive mobility variables and diffusion maps is available at https://zenodo.org/records/10846516 [40]. The source data of anonymous users’ mobile checking-in are accessible through a purchased licence to access China Unicom’s server. We used SQL queries to aggregate the individual traces to the locations’ 1665 mobility variables, which are accessible from the Zenodo project. The detailed mobile phone data are confidential for individual privacy reasons. We obtained access to the mobile phone data through China Unicom’s Local Area Network. A mobility variable dataset, which researchers can use to reproduce the results, is accessible at https://zenodo.org/records/10846516 [40]. Supplementary material is available online [41].
